# Antiatherogenic and Anti-Ischemic Properties of Traditional Chinese Medicine Xinkeshu via Endothelial Protecting Function

**DOI:** 10.1155/2012/302137

**Published:** 2011-10-10

**Authors:** Xu Tao, Peng Jing-bo, Zhang Wen-tong, Zhao Xin, Zhang Tao-tao, Yang Shi-jun, Fang Lei, Zou Zhong-mei, Cai Da-yong

**Affiliations:** ^1^Institute of Medicinal Plant Development, Chinese Academy of Medical Science and Peking Union Medical College, Beijing 100193, China; ^2^Neurology Department, China Meitan General Hospital, Beijing 100028, China; ^3^Neurology Department, Beijing Huimin Hospital, Beijing 100054, China

## Abstract

Including herbal medicine, complementary and alternative medicine (CAM) is popular worldwide. The traditional Chinese medicine xinkeshu has been widely used to treat coronary heart disease in China. This study was designed to investigate the protective effect and probable mechanism of xinkeshu tablet to atherosclerotic myocardial ischemia rabbit. Rabbits were divided into four groups (*n* = 12 each) and fed with different diet for 12 weeks: Control (standard diet), Model (high-cholesterol diet), XKS (high-cholesterol diet with 184.8 mg/kg/d xinkeshu), and Atorvastatin (high-cholesterol diet with 5.0 mg/kg/d atorvastatin). Plasma lipoprotein, ECG, endothelium-dependent vessel relaxation, histomorphological study, and expressions of eNOS and VCAM-1 on coronary arteries were assessed. The findings showed that, similar to atorvastatin, xinkeshu presented significant effects on rescuing endothelium-dependent vessel relaxation, inhibiting atherosclerotic progress, preventing myocardial ischemia, and changing eNOS and VCAM-1 expression. However, xinkeshu showed no lipoprotein lowering effect in hypercholesterolemia rabbits. The results of the present study indicated that xinkeshu exerted potent antiatherogenic and anti-ischemic properties on atherosclerotic myocardial ischemia rabbit. An endothelial protecting effect may be involved in the mechanism other than antihyperlipidemic effect.

## 1. Introduction

Coronary heart disease (CHD) is caused by atherosclerosis mainly, and high cholesterol levels play an important role in the onset of this disease [1]. The causes of atherosclerosis appear to be lipid retention, oxidation, and modification, which provoke chronic inflammation at susceptible sites in the walls of all major conduit arteries [2]. Although much progress has been made in reducing mortality from CHD, this condition remains the leading cause of death throughout the world [3]. Statins are the most potent drugs in this area. Studies have revealed that statins may not only lower low-density lipoprotein (LDL), but also elevate high-density lipoprotein (HDL) and enhance vascular endothelial function [4, 5]. However, liver dysfunction and myolysis as side effects of statins caused some patients to withdraw from treatment [6]. Complementary and alternative medicine (CAM), including herbal medicine, is popular in the general population worldwide [7, 8]. A number of herbs or plants with potent therapeutic components have been investigated for their antihyperlipidemic, antioxidant, and antiatherosclerotic properties [9–14]. The use of herbal medicine for the treatment of various disorders including heart diseases has a long and extensive history. 

In China, Traditional Chinese herbal products with low side effects are of high interest as CAM therapy to CHD [15–17]. Traditional Chinese medicine (TCM) xinkeshu (XKS) in tablet is a compound prescription formulated according to the meridian theory of TCM and approved in 2005 by the State Food and Drug Administration of China as a treatment of angina pectoris and arrhythmia patients in the clinic. Here, we have further investigated the mechanism of treatment with XKS tablet to atherosclerotic rabbits. Outcome can be summarized as follows: blood plasma lipoprotein levels; ECG test, indicator of severity of myocardial ischemia [18]; endothelium-dependent vessel relaxation (EDVR); histomorphological studies; expressions of endothelial nitric oxide synthase (eNOS) and vascular cell adhesion molecule 1 (VCAM-1) on coronary arteries, markers of endothelial function [19, 20].

## 2. Materials and Methods

### 2.1. Drugs and Reagents

XKS tablets were from Wo Hua Pharmaceutical Co, CHN. Cholesterol was from Tian Qi Chemical Engineering Co, CHN. Atorvastatin was from Jia Lin Pharmaceutical Co, CHN. Vasopressin (VP), phenylephrine (PE), and acetylcholine (Ach) were from Sigma, USA. Polyclonal Immunohistochemical goat anti-rabbit eNOS and VCAM-1 antibodies were from Santa Cruz, USA. Streptavidin/peroxidase kit and biotinylated mouse anti-goat IgG were from Boster, China.

### 2.2. Animals and Experimental Design

Japanese big ear rabbits (2.25 ± 0.20 kg, aged 3 weeks, male) were purchased from the Laboratory Animal Institute of the Chinese Academy of Medical Science. They were single-housed under a 12 : 12 h light-dark cycle, temperature-(23 ± 2°C) and humidity (50% ± 10%) controlled specific pathogen-free environment, with water available *ad libitum*. All animal care and experimental protocols complied with the Animal Management Rules of the Chinese Ministry of Health, and the study was approved by the animal ethics committee of the Chinese Academy of Medical Sciences. Standard diet pellets and high-cholesterol ester (H-ChE) diet [21] pellets which contain 2% cholesterol (240–280 g/d) for rabbits were prepared by Beijing Scientific Animal Feedstuff Company.

**Figure 1:**
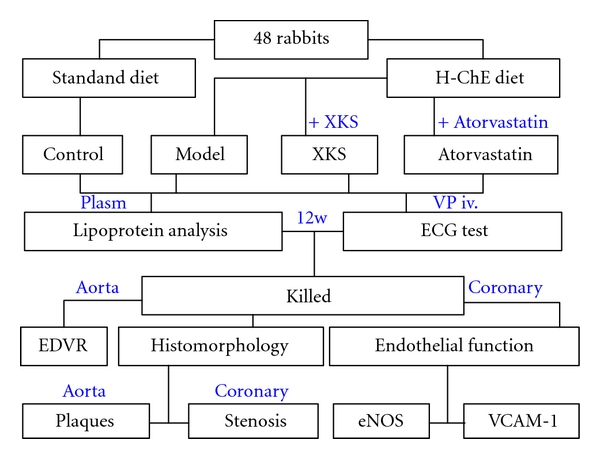
The experimental design. H-ChE, 2% cholesterol ester; XKS, Xinkeshu; VP, vasopressin; ECG, electrocardiogram; EDVR, endothelium-dependent vessel relaxation; eNOS, endothelial nitric oxide synthase; VCAM-1, vascular cell adhesion molecule 1.

Rabbits were divided into 4 groups (*n* = 12 per group) and the experimental design was presented in Figure 1.

**Control**

Rabbits were continuously fed with the standard pellets for 12 weeks. Rabbits were administrated intragastrically with normal saline (10 mL/kg/d).

**Model**

Rabbits were continuously fed with the H-ChE diet pellets. The others were the same as the Control.

**XKS**

Rabbits were administrated intragastrically with 184.8 mg/kg/d XKS (equivalent dose for an adult with mean weigh of 60 kg) in normal saline (10 mL/kg/d). The others were the same as the Model.

**Atorvastatin**

Rabbits were administrated intragastrically with 5.0 mg/kg/d atorvastatin in normal saline (10 mL/kg/d). The others were the same as the Model.

### 2.3. Plasma Lipoprotein Analysis

Fasting venous blood samples were collected in heparin from the marginal vein before and after the 12 weeks experiments. Plasma was separated and stored at −20°C. Plasma lipoprotein levels, including total cholesterol (TC), triglycerides (TG), LDL, and HDL were measured by use of an automatic biochemistry analyzer (Dimension AR, DuPont, USA).

### 2.4. ECG Test on VP-Induced Myocardial Ischemia Model

At the end of the 12-week experimental period, according to the method of Serradeil-Le Gal et al. [22], experimental of coronary vasospastic myocardial ischemia was induced by VP. The standard limb lead II of the ECG was recorded continuously before and 25 min after the administration of VP (2.0 IU/kg, *iv.*) with a Powerlab 30 system (AD Instruments, Castle Hill, Australia).

### 2.5. Assessment of EDVR

One week after the ECG tests were finished, rabbits (*n* = 6 per group) were anesthetized by 10% chloral hydrate (25 mg/kg, *ip*). According to the method of Lee et al. [23], the fresh hearts were immediately obtained and stored in cold PBS. Then the abdominal aortas were dissected and cut into 3 mm rings. The rings were stretched to 1.5 g tension and allowed to equilibrate for 60 min in a 10 mL tissue bath (38.6°C) containing Krebs-Henseleit solution (composition in mM: 115 NaCl, 25 NaHCO_3_, 1.38 NaH_2_PO_4_, 2.51 KCl, 2.46 MgSO_4_, 1.91 CaCl_2_, and 5.56 dextrose) and aerated with a mixture of 95% O_2_ and 5% CO_2_. Force generation was monitored by use of an isometric transducer connecting with the Powerlab 30 system. After equilibration, vasoconstriction was induced with 10^−6^ M PE. Once maximal contraction had reached a plateau, EDVR was determined as the response from 10^−9^ to 10^−4^ M Ach. The percent relaxation was calculated based on changes in the tension to the maximal precontraction value induced by PE.

### 2.6. Histomorphological Studies

The other rabbits (*n* = 6 per group) were anesthetized by 10% chloral hydrate (25 mg/kg, *ip*). Perfusion fixation was performed to every rabbit through the left common carotid artery with heparinized normal saline (70 mL/kg) and 4% paraformaldehyde (140 mL/kg) in 0.1 M phosphate buffer by use of an aortic catheter (at about 100 mmHg pressure), meanwhile the external jugular vein was cut for eliminating remained blood. Two hours later under 4.0°C, hearts and aortas were removed and immersion-fixed in 10% buffered formalin overnight.

The aortas were opened longitudinally along the posterior side and then stained with Sudan IV for visualization of the atherosclerotic plaques. After staining, the aortas were pinned open to flatten them and photographed. The total area (*A*_*T*_) and the plaques area (*A*_*P*_) of the aorta were morphometrically analyzed by use of Image-Pro Plus 7.0 morphometric analysis system (Media Cybernetics, USA). The atherosclerotic plaques ratio was calculated as *A*_*P*_ ÷ *A*_*T*_ × 100% [24].

Left circumflex coronary artery (2 cm long) with the adjacent myocardium tissues was carefully cut. Specimen were embedded in paraffin and cut into 5 *μ*m sections on a microtome, and the cross sections were then stained with hematoxylin and eosin (HE) and scanned by use of NanoZoomer Digital Pathology image analysis system (Hamamatus, Olympus, JAP). The area of the lumen (*A*_*L*_) and the area bordered as the internal elastic lamina (*A*_*I*_) were morphometrically analyzed by use of the Image-Pro Plus 7.0 analysis system for ×200 magnifications. The coronary stenosis ratio was calculated as (*A*_*I*_ − *A*_*L*_) ÷ *A*_*I*_ × 100% [25].

### 2.7. Immunohistochemical Studies of eNOS and VCAM-1 on Coronary Artery

eNOS and VCAM-1 expressions were evaluated immunohistochemically on coronary artery using the streptavidin/peroxidase kit according to the manufacturer's instructions. Sections were deparaffinized, rehydrated, and then soaked in antigen retrieval buffer (0.01 M Tris-base, 1.0 M EDTA, 0.05% Tween 20, pH 6.0) for 3 min at 95°C. Endogenous peroxidase activity was blocked by incubating the sections in 3% hydrogen peroxide aqueous solution for 1 h at room temperature. The sections were rinsed thrice with PBS, and then incubated with 100 *μ*L goat anti-rabbit eNOS or VCAM-1 antibody. The sections were rinsed with PBS and incubated with 100 *μ*L biotinylated mouse anti-goat IgG (1 : 100 dilutions in PBS). Protein was visualized with diaminobenzidine substrate solution. Primary antibody was substituted by PBS in the negative controls. The eNOS or VCAM-1staining area (*A*_*P*_) and observed area (*A*_*T*_) were morphometrically analyzed by use of the Image-Pro Plus 7.0 analysis system for ×400 magnification. The total existing eNOS or VCAM-1 was calculated semiquantitatively as *A*_*P*_ ÷ *A*_*T*_ × 100% [26].

### 2.8. Statistical Analysis

Statistical analyses involved use of SPSS, v13.0 (SPSS Inc., Chicago, IL, USA). Quantitative variables are expressed as means ± SEM. Comparison of continuous variables among multiple groups was performed by analysis of variance with ANOVA, and post hoc comparisons were made using LSD test.

## 3. Results

### 3.1. Plasma Lipoprotein Analysis

Before the 12-week experiment, the baseline values of plasma lipoprotein levels (TC, TG, LDL, and HDL) did not vary significantly among the four groups. After the 12-week experiment, Model group rabbits showed significant increment in the levels of TC (*P* < 0.01), LDL (*P* < 0.01), TG (*P* < 0.05) and significant reduction in the level of HDL (*P* < 0.05) compared with Control. Atorvastatin treatment for 12 weeks significantly reduced TC (*P* < 0.01), LDL (*P* < 0.01), and TG (*P* < 0.05) levels and significantly increased HDL (*P* < 0.05) level compared with Model. However, XKS treatment showed a slight reduction (*P* > 0.05) in the TC, TG, LDL levels and a slight increment (*P* > 0.05) in the levels of HDL compared with Model (Table 1).

### 3.2. ECG Test on VP-Induced Myocardial Ischemia Model

**Figure 2:**
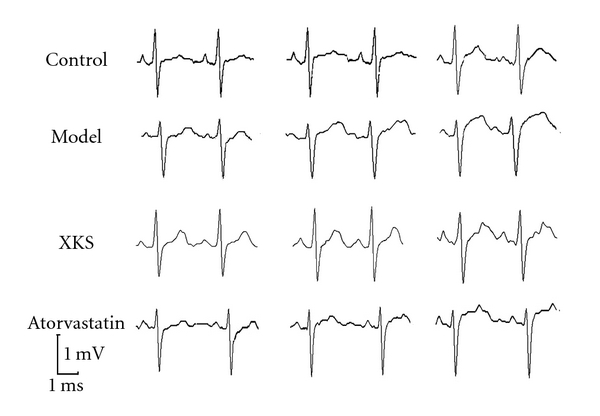
Max ST segment elevation in ECG after vasopressin administration.

Injection of VP (iv.) into conscious rabbits induced transient ST segment elevation in the ECG in each group. The maximum ST segment elevation was observed 5–10 min after VP administration in Control. Model group rabbits showed significantly higher (*P* < 0.01) ST segment elevation than Control. XKS treatment showed significant (*P* < 0.01) anti-ischemic effect (inhibition of ST segment elevation induced by VP) than Model. Atorvastatin also showed significant (*P* < 0.05) anti-ischemic effect; however, XKS was more effective (*P* < 0.05) than Atorvastatin (Table 2 and Figure 2).

**Figure 3:**
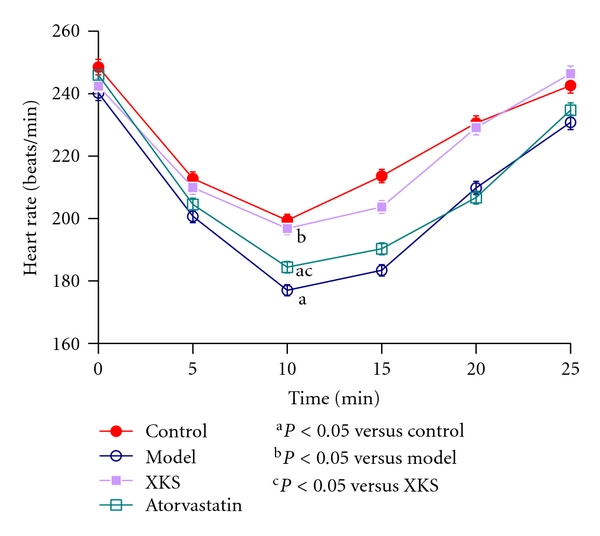
Heart rate curves after vasopressin administration.

Transient heart rate (HR) decrement occurred after VP administration in each group. The effect peaked after 10–15 min in Control. Model group rabbits showed more obvious HR decrement (*P* < 0.05) compared with Control. XKS treatment showed significant (*P* < 0.05) inhibition on HR decrement compared with Model. However, no significant inhibition effect (*P* > 0.05) was observed with atorvastatin treatment compared with Model (Figure 3).

### 3.3. Assessment of EDVR

**Figure 4:**
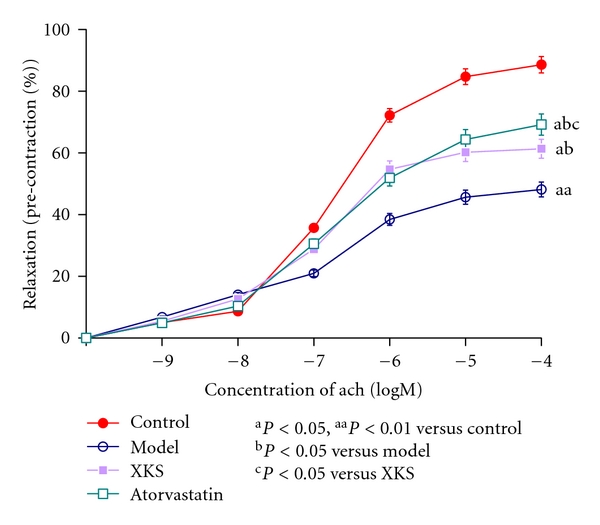
Endothelia-dependent vessel relaxation curves of abdominal aorta rings.

Ach (10^−9^ to 10^−4^ M) caused a concentration-dependent relaxation in preconstricted abdominal aorta rings. The max EDVR was significantly impaired (*P* < 0.01) in Model group rabbits compared with Control. XKS and Atorvastatin treatment significantly (*P* < 0.05) attenuated the impairment compared with Model. Atorvastatin was more effective (*P* < 0.05) than XKS (Figure 4).

### 3.4. Histomorphological Studies

**Figure 5:**
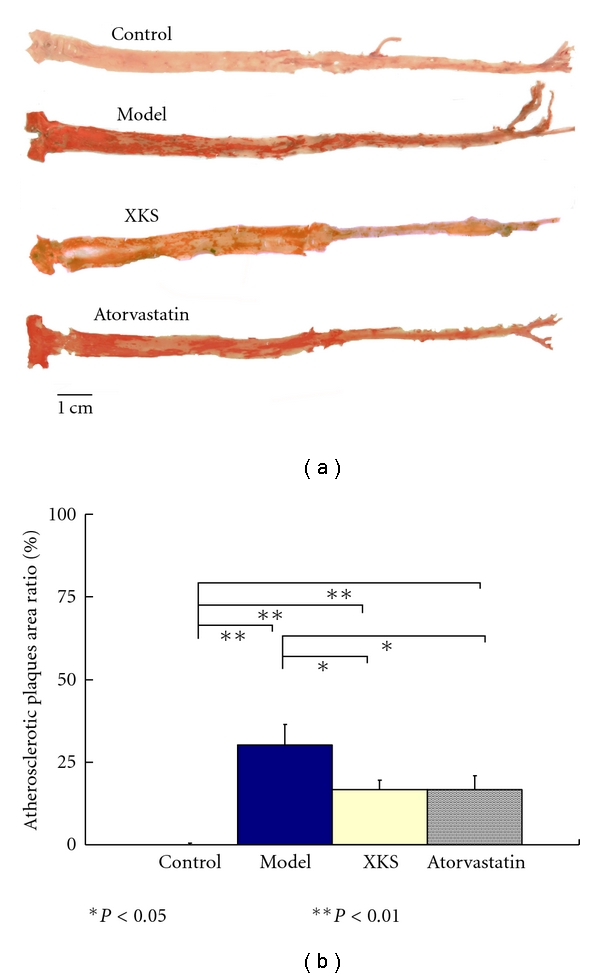
Atherosclerotic plaques on the intimal surface of aorta by Sudan IV staining.

None of Control group rabbits showed any abnormal histological changes in the aorta. Typical macroscopic atherosclerotic plaques on the intimal surface of aortas can be seen distinctly and commonly in Model rabbits. Atherosclerotic plaques became red color by Sudan IV staining. XKS and Atorvastatin treatment significantly (*P* < 0.05) reduced the atherosclerotic plaques area compared with Model. The effect was similar (*P* > 0.05) between the two groups (Figure 5). 

**Figure 6:**
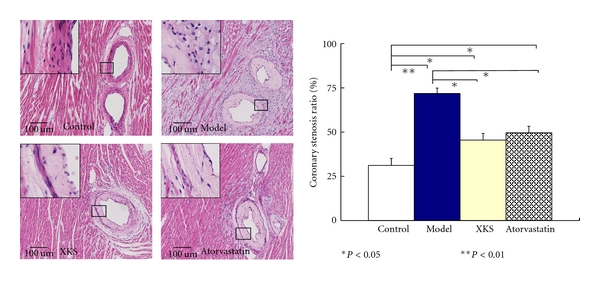
Coronary stenosis by HE staining (light micrographs, middle 100×, left top 400×).

No atherosclerotic changes of any arterial wall were presented in Control group rabbits. But in Model rabbits, some intramyocardial small arterioles showed significant atherosclerotic changes, including that the basal laminae around the smooth muscle cells were irregularly thickened and multilaminated. The collagen fibrils had significantly increased in the media, and a large number of lipids had infiltrated into the thickened intima. The coronary lumens became stenosis accompanied with lipids deposition that contained foam cells. Model group rabbits showed significant (*P* < 0.01) coronary stenosis than Control. XKS and Atorvastatin treatment significantly (*P* < 0.05) inhibited the coronary stenosis compared with Model. The effect was similar (*P* > 0.05) between the two group (Figure 6).

### 3.5. Immunohistochemistry Studies of eNOS and VCAM-1 on Coronary Artery

**Figure 7:**
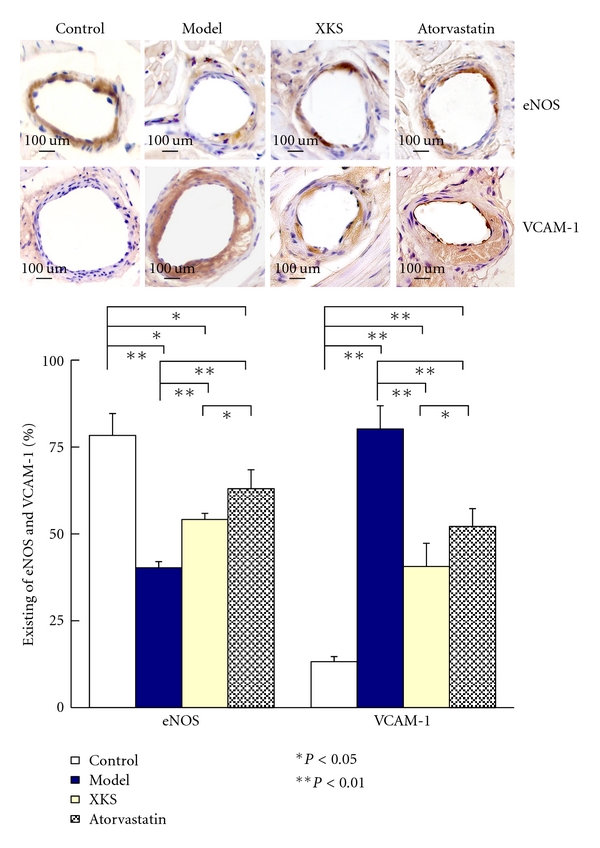
Existing of eNOS and VCAM-1 on coronary artery by immunohistochemistry staining (light micrographs, 400×).

In Control group, the eNOS positive staining could be observed in the cytoplasm of coronary intimal layer area. The existing of eNOS was significantly decreased (*P* < 0.01) in Model group compared with Control. XKS and Atorvastatin treatment significantly (*P* < 0.01) increased the existing eNOS compared with Model. Atorvastatin was more effective (*P* < 0.05) than XKS (Figure 7).

In Control group, VCAM-1 positive staining was seldom observed in the entire wall of coronary artery. However, it could be observed largely in collagen fibrils and foam cells rich area of the vascular wall in Model group, and the existing of VCAM-1was significantly (*P* < 0.01) increased than Control. XKS and Atorvastatin treatment significantly (*P* < 0.01) decreased the existing of VCAM-1 compared with Model. XKS was more effective (*P* < 0.05) than Atorvastatin (Figure 7).

## 4. Discussion

CAM including herbal medicine has gained a worldwide popularity over the past 20 years. It is argued that patients with chronic conditions including cardiovascular disease are likely to use CAM [27, 28]. Herbal medicine is the method with the use of medicinal plants or herbs for prevention and treatment of diseases, and it ranges from traditional and popular medicines of every country to the use of standardized and titrated herbal extracts [29].

 XKS tablet was being widely used to treat CHD by the traditional practitioners in China over ten years [30]. Clinical research revealed that XKS carried many biological activities, including improving of heart rate variability, reducing the episode of angina pectoris, improving the arterial elasticity [31, 32]. Meanwhile, pharmacological basic research revealed that XKS administration had a variety of therapeutic effects such as decreasing myocardial oxygen consumption, lowering lipid, and antiapoptosis [33–35]. 

In the present study, atorvastatin was chosen as a positive control therapy. The findings showed that atorvastatin treatment for 12 weeks was very effective in lowering the plasma TC and LDL levels, increasing HDL level, lessening experimental myocardial ischemia, rescuing EDVR, and inhibiting atherosclerotic progress. XKS treatment for 12 weeks presented the similar effects on rescuing EDVR and inhibiting atherosclerotic progress as atorvastatin did. Even XKS was more effective on preventing myocardial ischemia and maintaining the cardiac rhythm than atorvastatin. Maybe these properties were the main mechanisms of XKS for clinical angina pectoris and arrhythmia therapy [31, 32]. On the other hand, one of the important findings in the present study was that no significant changes in lipid profiles occurred in the rabbits administered with XKS. In other words, XKS showed no lipoprotein lowering effect to the hypercholesterolemia induced by H-ChE diet. 

It is well known that endothelial injury is a key event in the pathogenesis of atherosclerosis. Atherosclerosis can be induced from simple dysfunction of endothelial lining as occurs with hypercholesterolemia [36]. Endothelial cell homeostasis is maintained largely through the synthesis of nitric oxide (NO), a potent vasodilator synthesized by eNOS. NO serves important functions, including regulation of vascular tone and regional blood flow and suppression of vascular smooth muscle cell proliferation. eNOS is affected by different stimuli, including hypoxia, shear stress, LDL, and the development and progression of atherosclerosis [37]. The decreasing expression or inactivation of eNOS is recognized to be a crucial factor in the development of endothelial dysfunction [19]. The important role of vascular adhesion molecules in atherosclerosis has been discovered and these molecules play an important role in adhesion of circulating leukocytes to endothelium, which is the first step in initiation of atherosclerosis [38]. As a transmembrane glycoprotein, VCAM-1 is upregulated and expressed at atherosclerosis-prone sites even before macroscopic disease is apparent, with persistent expression in more advanced atherosclerotic lesions. Atherogenic diet could rapidly induce VCAM-1 expression in aortic endothelium in aortic organ cultures [20]. 

According to the novel property of antiatherogenic and antiischemia not via antihyperlipidemia pathway, we focused on endothelial protection as the target to investigate the indeed mechanism of XKS. eNOS and VCAM-1 were picked as antiatherogenic and atherogenic factors, respectively. The findings showed that the 12 weeks of H-ChE diet caused decreasing expression of eNOS as well as increasing expression of VCAM-1during the procedure of atherosclerosis. Atorvastatin and XKS treatment both showed vascular protecting property by changing the expression of eNOS and VCAM-1. Therefore, significant endothelia protection to vascular was probably one of the important mechanisms involved in cardioprotective properties of XKS. 

XKS includes 5 herbal medicinal components, and they are *Salvia miltiorrhiza Bunge*, *Panax notoginseng* (PN), *Fructus Crataegi*, *Radix Puerariae*, and *Radix Aucklandiae* (Table 3). Materials were originally ground to a fine powder by a micronizer and prepared as tablet, which were authenticated and standardized on the basis of marker compounds in the Chinese Pharmacopoeia 2010 [39]. Several groups of monomer with special biological activities were extracted from each single component, for example, attenuating pulmonary fibrosis of PN extract [40], lowering plasma cholesterol of *Hawthorn* extract [41], improving insulin resistance of *Puerarin* extract [42], and attenuating idiopathic edema of *radix Aucklandiae* extract [43]. According to the theory of TCM, *Salvia miltiorrhiza Bunge* was looked on as a kind of “principal drug”, *Panax notoginseng* as “ministerial drug”, and the other 3 components served as “adjunctive drug” among components of XKS [44]. Tanshinone IIA was one of the most important monomer ingredients of Salvia miltiorrhiza Bunge extract [45]. The biological activities of Tanshinone IIA were about decreasing myocardial oxygen consumption [46], dilation of coronary arteries [47], and improving neuron regeneration [48], antihypertension [49], and antioxidant [50]. 

In the present study, we looked on XKS as a single “medicine” and investigated the pharmacological action of all components together. Although the results of present study provided impetus for further studies on the therapeutic action of XKS, the relationship of these components and their interactions remained to be clarified. Those were the main limitations of the present study. Therefore, the detailed molecular mechanism of XKS and further studies in animals about the pharmacological of the active ingredients and metabolites should be investigated.

## 5. Conclusion

In conclusion, it was explicitly demonstrated that TCM XKS exerted potent antiatherogenic and anti-ischemic properties on the atherosclerotic myocardial ischemia rabbit model. An endothelial protecting effect may be involved in the mechanism other than antihyperlipidemic effect. We believed that a better understanding of the mechanisms by which XKS protecting endothelia and the interactions of active ingredients could lead to novel pharmacological CAM interventions for CHD patients.

## Figures and Tables

**Table 1:** Plasma lipoprotein levels before and after 12 weeks period experiment.

Parameters	Before				After			
(mmol/L)	Control	Model	XKS	Atorvastatin	Control	Model	XKS	Atorvastatin
TC	1.37 ± 0.13	1.18 ± 0.12	1.24 ± 0.08	1.29 ± 0. 11	1.43 ± 0.05	27.83 ± 2.43^aa^	26.60 ± 0.30^aa^	17.19 ± 1.54^aabbcc^
TG	0.92 ± 0.18	1.01 ± 0.26	0.88 ± 0.12	0.95 ± 0.13	0.52 ± 0.03	2.11 ± 0.17^a^	1.58 ± 0.05^a^	0.69 ± 0.06^bc^
LDL	0.59 ± 0.01	0.64 ± 0.06	0.54 ± 0.08	0.57 ± 0.09	0.45 ± 0.01	15.11 ± 2.74^aa^	15.06 ± 2.16^aa^	7.63 ± 1.22^aabbcc^
HDL	3.54 ± 0.05	4.05 ± 0.06	3.81 ± 0.04	3.30 ± 0.02	3.49 ± 0.04	2.47 ± 0.15^a^	2.48 ± 0.16^a^	3.17 ± 0.15^abc^

Data are expressed as mean ± SEM, *n* = 12, ^a^ *P* < 0.05  ^aa^ *P* < 0.01 versus Control; ^b^ *P* < 0.05  ^bb^ *P* < 0.01 versus Model; ^c^ *P* < 0.05  ^cc^ *P* < 0.01 versus XKS.

**Table 2:** ST segment elevation (mV) on ECG after vasopressin administration.

Group	Time (min)					
	2	5	10	15	20	25
Control	0.08 ± 0.01	0.19 ± 0.04	0.24 ± 0.05	0.17 ± 0.06	0.04 ± 0.01	0.02 ± 0.00
Model	0.07 ± 0.02	0.40 ± 0.12^aa^	0.56 ± 0.12^aa^	0.33 ± 0.08^a^	0.25 ± 0.12^aa^	0.07 ± 0.01
XKS	0.08 ± 0.01	0.27 ± 0.08^ab^	0.37 ± 0.10^aabb^	0.20 ± 0.10^b^	0.12 ± 0.10^ab^	0.04 ± 0.00
Atorvastatin	0.06 ± 0.01	0.38 ± 0.15^abc^	0.46 ± 0.11^aabc^	0.26 ± 0.09^a^	0.16 ± 0.06^ab^	0.06 ± 0.01

Data are expressed as mean ± SEM, *n* = 12, ^a^ *P* < 0.05  ^aa^ *P* < 0.01 versus Control; ^b^ *P* < 0.05  ^bb^ *P* < 0.01 versus Model; ^c^ *P* < 0.05 versus XKS.

**Table 3:** Formulation of xinkeshu tablet.

Latin binomial	Herb or plant sources	Part used	Portion (%)
Radix salviae miltiorrhiae	Salvia miltiorrhiza Bge.	Root and rhizome	32
Panax notoginseng	Panax Notogin seng (Burk) F.H Chen	Root and rhizome	2
Hawthorn	Crataegus pinnatifida Bge.	Fruit	32
Radix Puerariae	Pueraria lobata	Root and rhizome	32
Radix Aucklandiae	Aucklandia lappa Decne.	Root and rhizom	2
